# Providing live black soldier fly larvae (*Hermetia illucens*) improves welfare while maintaining performance of piglets post-weaning

**DOI:** 10.1038/s41598-021-86765-3

**Published:** 2021-04-01

**Authors:** Allyson F. Ipema, Eddie A. M. Bokkers, Walter J. J. Gerrits, Bas Kemp, J. Elizabeth Bolhuis

**Affiliations:** 1 Adaptation Physiology Group, Department of Animal Sciences, Wageningen University & Research, P.O. Box 338, 6700 AH Wageningen, The Netherlands; 2 Animal Production Systems Group, Department of Animal Sciences, Wageningen University & Research, P.O. Box 338, 6700 AH Wageningen, The Netherlands; 3 Animal Nutrition Group, Department of Animal Sciences, Wageningen University & Research, P.O. Box 338, 6700 AH Wageningen, The Netherlands

**Keywords:** Behavioural methods, Animal behaviour, Animal physiology

## Abstract

During weaning, piglets experience concurrent social, physical, and nutritional stressors. Consequently, piglets often have poor feed intake and display increased oral manipulative behaviours post-weaning, indicative of compromised welfare. Black soldier fly larvae (BSFL) possess many attractive properties for pigs and could therefore function as effective edible enrichment, potentially alleviating weaning stress by facilitating exploration and promoting feed intake. In this study, pairs of piglets received a small amount of either live BSFL or wood shavings (8 pens/treatment) scattered throughout the pen twice a day for 11 days after weaning. Home-pen behaviour was scored by instantaneous scan sampling on day 2, 5 and 8, and behavioural responses to a novel environment and novel object were scored on day 10/11. Performance-related parameters were observed regularly. Larvae provisioning increased floor-directed exploration and decreased object-directed exploration, pig-directed oral manipulation, fighting and eating of pellets, and reduced neophobia towards a novel object. Pellet intake was significantly decreased by BSFL provisioning during day 4–11 post-weaning, although feed and net energy intake including BSFL never differed between treatments. BSFL provisioning did not influence piglet growth, feed efficiency, energy efficiency, and faecal consistency. To conclude, live BSFL provisioning positively affected post-weaning piglet behaviour while maintaining performance.

## Introduction

On commercial pig farms, piglets are often weaned abruptly around 3–4 weeks of age. This process involves several stressors, including separation from the sow, transitioning from a mainly liquid (milk) diet to a completely solid diet, and usually relocation to a new environment and mixing with unfamiliar piglets. Piglets are generally neophobic towards unfamiliar feed items^1^, and early and abrupt weaning often results in decreased feed consumption post-weaning. Decreased feed consumption can reduce growth and intestinal integrity, and increase disease susceptibility, reflected by the high incidence of post-weaning diarrhoea (as reviewed by^2,3^). Furthermore, abruptly weaned piglets frequently display increased levels of aggression and pig- and pen-directed oral manipulation behaviour, especially when housed in barren environments^4,5^. Early and abrupt weaning can severely impact the welfare of piglets, and may have lifelong detrimental effects on pig performance and intestinal health^6,7^.

One approach to ease the weaning transition would be to implement systems that allow for more gradual weaning, such as group farrowing systems with a prolonged lactation period^8^, though this requires large-scale adjustments to current husbandry management. Alternatively, providing environmental enrichment post-weaning can benefit piglet welfare, as its presence may facilitate enrichment oriented exploration, reducing the motivation to explore and manipulate pen mates^4^. Furthermore, previous studies have indicated that enriched-housed piglets often respond less neophobic towards novel objects^9^ and novel feed items^10^ than barren-housed piglets. Hemsworth et al. attributed reduced neophobia to regular confrontation with novel items which likely habituates pigs to novelty^11^. Additionally, stressful situations were found to increase food neophobia in mammals such as sheep and humans^12,13^. For pigs, it was found that environmental enrichment can reduce stress^14^, and as such enrichment could lessen feed neophobia. Reduced feed neophobia may result in increased feed intake^10^ and consequently better performance post-weaning. It has been shown, for example, that providing hanging ropes and tyres to piglets during several weeks after weaning improved piglet growth and reduced fearful responses towards a person^15^, and providing hanging ropes and wood shavings also improved piglet growth and increased exploration behaviour^16^. Other studies demonstrated that providing peat (for 5 weeks post-weaning) or providing increased space combined with straw, peat and branches (for 2 weeks post-weaning) reduced pig-directed oral manipulation behaviour, and increased exploration behaviour and growth^4,17,18^.

Edible items (e.g. straw) are very effective as enrichment, as they provide a consumable reward and thereby reinforce exploration behaviour^19^. Exploring and eating edible enrichment allow piglets to develop feeding-related behaviours such as chewing, adapting them to consuming solid feeds^20^, while simultaneously benefitting gut functioning^21^. Furthermore, providing edible enrichment creates dietary diversity, which has been shown to increase feed intake before weaning and may have a similar effect after weaning^22,23^. Durán et al. found that newly weaned piglets actively interact with and consume whey-based edible enrichment^24^. Also, providing straw post-weaning increased exploration behaviour and reduced pig- and pen-directed oral manipulation^25,26^. These studies highlight the potential of using edible enrichment post-weaning to ease the weaning transition, though this has not been widely studied.

Living insect larvae, which are a natural feed source of wild boar^27^, have the potential to function as effective edible enrichment. One species suitable for consumption by commercial pigs is the black soldier fly. Black soldier fly larvae (BSFL) are rich in fat, calcium, and protein with an appropriate amino acid profile for pigs^28–30^. Their palatability and high moisture content (55–65%)^28,29,31^ might stimulate intake by newly weaned piglets that are accustomed to a liquid milk diet, and their consistency could allow larvae to function as a transitory feed between milk and concentrate. BSFL are essentially free of carbohydrates^28^, therefore digestion is not limited by low secretion of starch-degrading enzymes in recently weaned piglets. In addition, BSFL possess many characteristics of effective enrichments. The larvae are not only edible, but also odorous, destructible, and manipulable, and these properties are highly interesting for pigs and facilitate exploration behaviour^32,33^. The ability of live larvae to crawl to different places could decrease the predictability of interacting with the larvae, and decreased predictability is known to prolong interest in enrichment^34^. Moreover, piglets seem to prefer interacting with larger sized feed items. Previous studies found that piglets prefer interacting with 5-mm diameter feed pellets over smaller 1.8-mm diameter pellets^35^, and they prefer interacting with 12-mm diameter pellets over 2-mm diameter pellets^36^. BSFL can grow up to 6 mm wide and up to 15–27 mm long^28,37,38^, and are thus substantially larger than most commonly provided pelleted feeds. Like other enrichment items, the presence of larvae might habituate pigs to novelty and/or have stress-reducing effects on pigs, potentially reducing neophobia.

Altogether, the properties of live BSFL suggest that they could function as effective edible enrichment post-weaning. This study aims to explore this potential by determining the effect of live BSFL provisioning post-weaning on piglet behaviour, fearfulness and neophobia, growth, feed and energy intake, feed and energy efficiency, and faecal consistency. It is expected that live BSFL provisioning will promote behaviours that are indicative of good welfare, e.g. exploration and play behaviours, and reduce aggression and pig- and pen-directed oral manipulation behaviour. Furthermore, providing live BSFL is expected to reduce neophobia and improve the performance and faecal consistency of post-weaning piglets.

## Methods

The experimental protocol was approved by the Animal Care and Use committee of Wageningen University & Research under project licence number AVD1040020187184. The protocol was in accordance with the Dutch animal experimentation law and complies with European Directive 2010/63/EU. The experiment was carried out at the animal experiment facilities of Wageningen University & Research (Wageningen, The Netherlands). The ARRIVE guidelines for reporting animal experiments were taken into account in this study^39^.

### Animals, housing and management

A total of 32 female, tail docked piglets (Pietrain x TN70) from 15 litters (range parity sows: 1–5) were weaned at 24.8 ± 0.8 days of age (day 0) and transported to the experimental facility. Pairs of unfamiliar piglets were selected based on body weight, to achieve a similar average weight (7.4 ± 0.2 kg) per pen. Pens were distributed over two rooms, balanced for treatment. Pairs of pigs were housed in pens of 2.85 × 1.20 m equipped with a feed trough (12 × 50 cm with three feeding places), one drinking nipple and one rubber ball on a chain. Feed and water were provided ad libitum. Flooring of all pens was a rubber mat covered by a 5 cm layer of wood shavings. Pigs were fed the creep feed they had also received during the suckling period (*Speenkruimel Fit*, ABZ Diervoeding) until day 2 after weaning. This feed was then mixed with the weaner feed (*Speen Havic Top*, AgruniekRijnvallei) for one day, after which only the weaner feed was provided. As feed consumption was initially very low, in all pens a spoonful of sugar was added when the weaner diet was first provided to ease the feed transition. In the experimental rooms a radio and lights were on from 07:00 to 19:00 h, and between 19:00 and 07:00 h the lights were dimmed, and the radio was off. Room temperature was 27 °C on arrival and was gradually decreased to 24 °C on day 11 post-weaning (the end of the experiment).

### Experimental design

Pens were assigned to either the control (CON) or the black soldier fly larvae (LAR) treatment (8 pens/treatment). Piglets were provided with wood shavings (CON) or live BSFL (LAR) twice a day, at 09:00 and 13:00 h, from day 1 after weaning, by scattering the item in the pen on the wood shavings bedding. Wood shavings were provided to the CON treatment to create a similar disturbance in all pens. LAR pens received either 75 g (day 1–4) or 150 g (day 5–11) of live larvae twice a day, and CON pens always received a similar volume of wood shavings (approximately 100 and 200 ml, respectively). Live, 14-day-old BSFL were supplied weekly (by Bestico B.V., Berkel en Rodenrijs, The Netherlands) and stored at 12 °C. From approximately 30 min before provisioning, larvae were stored at room temperature to increase larvae activity.

### Measurements

#### Home-pen behaviour

At arrival and on day 7 of the experiment, each piglet was marked with a colour stripe (stock marker spray) on their back for individual identification. The behaviour of all pigs was observed by 1-min interval instantaneous scan sampling on day 2, 5 and 8 post-weaning according to the ethogram in Table 1. Each of these 3 days had seven observation periods, starting at 07:15, 09:00, 10:15, 11:30, 13:00, 14:15 and 17:00 h. The periods starting at 09:00, 10:15, 13:00 and 14:15 h were observed live, where two observers observed 8 experimental pens (4 CON and 4 LAR) in one room each, switching pens every hour. Before observing, the observers were trained, and inter-observer reliability was regarded “almost perfect” (Cohen’s kappa > 0.8)^40^. The remaining observation periods were observed afterwards from video by one person (one of the two observers). All observations were done using the program Observer 14.2 (Noldus Information Technology B.V., Wageningen, The Netherlands).

**Table 1:** Ethogram of behaviours observed in the home pen.

Behaviour	Description
Pig-directed oral manipulation	Nosing, rooting, sucking, nibbling, or chewing body of pen mate, or belly nosing (rubbing belly of a pen mate with up and down snout movements)
Fighting	Pushing, pressing, ramming, head knocking, nudging, or lifting pen mate, can include aggressive biting
Social play	Group wise running, jumping, rolling, and/or turning in the pen
Non-social play	Running, jumping, or turning in the pen individually, and/or shaking head while holding toy/bedding, throwing bedding in the air
Exploring floor	Sniffing, touching, rooting, or chewing the floor or bedding material, potentially including larvae
Exploring object	Sniffing, touching, rooting, or chewing walls, feeders, or toys (above floor level)
Eating feed	Having head in feeder while eating or manipulating feed
Inactive	Sitting or lying, without performing any other behaviour
Other	Performing any other behaviour

#### Novel environment and novel object test

To determine pig responses to novelty, all experimental pigs, except one pig from the LAR treatment which was lame on the day of testing, were subjected to a Novel Environment Test and subsequent Novel Object Test. Tests were performed on day 10 and 11 post-weaning between 09:00 and 15:00 h. Half of the pigs were tested on 1 day and the other half on the other day, with the order being balanced for treatment and room. Prior to testing, pigs were individually caught, placed in a closed cart, and transported to an experimental room nearby. Here, the pig was placed in a start box alongside the experimental area. The door of the start box was opened within 10 s, giving the pig access to an unfamiliar area of 5.3 × 5.3 m with a rubber floor, surrounded by 1 m high hardwood walls. The novel environment test started when the pig had entered the area and the door was closed behind it. After 5 min, the novel object test was started by lowering an unfamiliar object (a metal bucket) from the ceiling in the centre of the area. The bucket was placed on the floor, which resulted in a noise. The rope attached to the bucket was held loosely. After five more minutes the test ended, and the pig was transported back to the home pen. During the tests, two observers were in the same room but out of sight of the pig, viewing the pig on a screen. One observer scored the behavioural and postural states, and the other observer scored behavioural events, according to the ethogram in Table 2. Scoring was done on a tablet with the program Observer 14.2 (Noldus Information Technology B.V., Wageningen, The Netherlands). Between tested pigs, faeces and urine were removed and the experimental area was cleaned with water and soap and dried with towels.

**Table 2:** Ethogram of behaviours observed in the novel environment test and novel object test.

Item	Description
**Exclusive behavioural states**	
Exploring environment	Sniffing, nosing, rooting, chewing, or licking the floor or wall
Exploring object	Sniffing, nosing, rooting, chewing, or licking the object
Drawing back	Quickly moving a few steps away from the object after being oriented towards the object
Other	Performing any other behaviour
**Exclusive postural and locomotive states**	
Moving	Walking or running
Freezing	Standing motionless on four legs with head fixed (up or down) and ears upright
Standing	Standing motionless on four legs, not alert
Sitting	Sitting or kneeling on the floor
Lying	Lying with side or belly touching the floor
**Behavioural events**	
Defecating	Excreting faeces
Urinating	Excreting urine
High-pitched vocalizations	Screams, squeals, or grunt-squeals
Low-pitched vocalizations	Short or long grunts

#### Performance

Piglets were individually weighed directly after arrival at the experimental facilities, and on day 1, 4, 7 and 11 post-weaning. Pellet intake per pen was determined by weighing leftovers on day 1, 2, 4, 7 and 11. Regular observations of the bedding indicated that live larvae were almost absent within a few hours after provisioning, therefore complete consumption of the larvae was assumed. Prior to the experiment, three samples of BSFL were analysed for their dry matter (DM), crude protein, crude fat, calcium, and phosphorus content, and based on this the net energy content (MJ NE/kg DM) of the larvae was calculated (Supplementary Table S1). The net energy content of the pelleted feed (MJ NE/kg DM) was calculated using the energy values provided by the feed manufacturers. The DM intake per pen excluding and including larvae were calculated and analysed separately, and based on the total DM intake including larvae the net energy intake (MJ NE), feed efficiency (weight gain (g)/dry matter intake (g)), and energy efficiency (weight gain (g)/net energy intake (MJ NE)) were calculated to account for the large difference in the net energy content between pellets and BSFL.

#### Faecal consistency and dry matter content

Every day at 08:30 h the faecal consistency of individual pigs was scored by two observers. Faecal consistency scores as visible around the anus of the pig at that time were based on the definition by Pedersen and Toft^41^. Score 1 represents firm faeces, score 2 soft but shaped faeces, score 3 loose faeces and score 4 water thin faeces. On day 4 and 7 faecal samples were collected during the weighing procedure, either by allowing the piglet to defecate by itself, or by stimulating defecation through gentle anal manipulation with a cotton swab for at most 10 s. The faecal samples were weighed before and after drying at 70 °C for 24 h and based on this the dry matter percentage was calculated.

### Statistical analyses

#### Data processing

The feeder in one CON pen had fallen over on the last experimental day and this data was excluded from analysis. Home-pen behaviours were summed per piglet per day and expressed as the percentage of observed scans. To limit the number of variables, a factor analysis was conducted on data from the novel environment test and the novel object test (e.g.^42^). Behavioural states and postures were expressed as a percentage of time, and behavioural events were expressed as absolute frequencies. During the Novel Environment and Novel Object Test the behaviours “drawing back”, “defecating”, and “urinating”, and the postures “sitting” and “lying” were very rare, and they were not included in the factor analysis. The behaviours “high-pitched vocalizations” and “low-pitched vocalizations” were combined into “vocalizations” for the factor analyses. The latency (s) to explore the novel object was calculated and included in the factor analysis. Prior to analysis, a Pearson’s correlation test performed on all variables showed that the variable “standing” was strongly negatively correlated to “moving” in the novel object test (r =  − 0.80), therefore “standing” was excluded from the factor analysis. For the novel environment test, the variables “freezing” and “moving” each contained one outlier (based on the Grubb’s test), and these outliers were excluded from analysis. The variables “freezing” (for the novel environment test and the novel object test) and “exploring object” (for the novel object test) were arcsine square root transformed, and “latency to explore object” was logarithmically transformed for normalization.

#### Data analysis

All data were analysed using the statistical software SAS 9.4 (SAS Institute Inc., Cary, NC, USA). General linear (mixed) model residuals were checked for normality.

The proportions of time spent on each observed behaviour in the home pen were analysed in generalised linear mixed models (GLIMMIX in SAS) with a binomial distribution, logit link function, and additional multiplicative over-dispersion parameter. Treatment, room, and day were included as fixed effects. The interaction between day and treatment was removed from the final models as this effect was never significant. Furthermore, the models included a random effect of pen nested within treatment and room, and a repeated effect of test day with pig as subject, using a homogenous first-order autoregressive covariance structure.

The variables obtained from the novel environment test and the novel object test were put in a factor analysis with orthogonal varimax rotation, and factors with an eigenvalue above one were retained. This resulted in one factor for the novel environment test and one factor for the novel object test (Table 3), explaining 63% and 72% of the variation, respectively. The scores of each pig for each factor were analysed with a linear mixed model (MIXED in SAS) including a fixed effect of treatment, room and test day and a random effect of pen nested in treatment and room.

**Table 3:** Loadings of the factors with an eigenvalue above one that were extracted by factor analysis with orthogonal varimax rotation on the behaviours and postures scored during the novel environment test (NET) and the novel object test (NOT).

Variable	NET factor 1	NOT factor 1
	“Fearful”	“Neophobic”
Exploring environment (% of time)	** − 0.57**	− 0.34
Moving (% of time)	** − 0.93**	** − 0.67**
Standing (% of time)	**0.76**	
Freezing (% of time)	**0.60**	**0.81**
Vocalizations (frequency)	**0.56**	− 0.26
Exploring object (% of time)		** − 0.69**
Latency to explore object (s)		**0.76**
Eigenvalues	2.45	2.35
% of variance explained	63%	72%

High loadings (loadings ≤  − 0.5 or ≥ 0.5) are indicated in bold.

Body weight and average daily gain were analysed in a linear mixed model including a fixed effect of treatment and room, a random effect of pen nested in treatment and room, and weight directly after weaning as a covariate. Dry matter intake excluding and including BSFL, net energy intake, feed efficiency, and energy efficiency were analysed for treatment effects on pen level in general linear models. The proportion of faecal dry matter was analysed per day in a similar model as the home-pen behaviours but without a repeated effect.

P-values below 0.05 were considered statistically significant. Significant fixed effects were further analysed using differences in least square means with a Tukey’s HSD correction. Data are presented as pen means ± SEM unless stated otherwise.

## Results

### Home-pen behaviour

**Figure 1:**
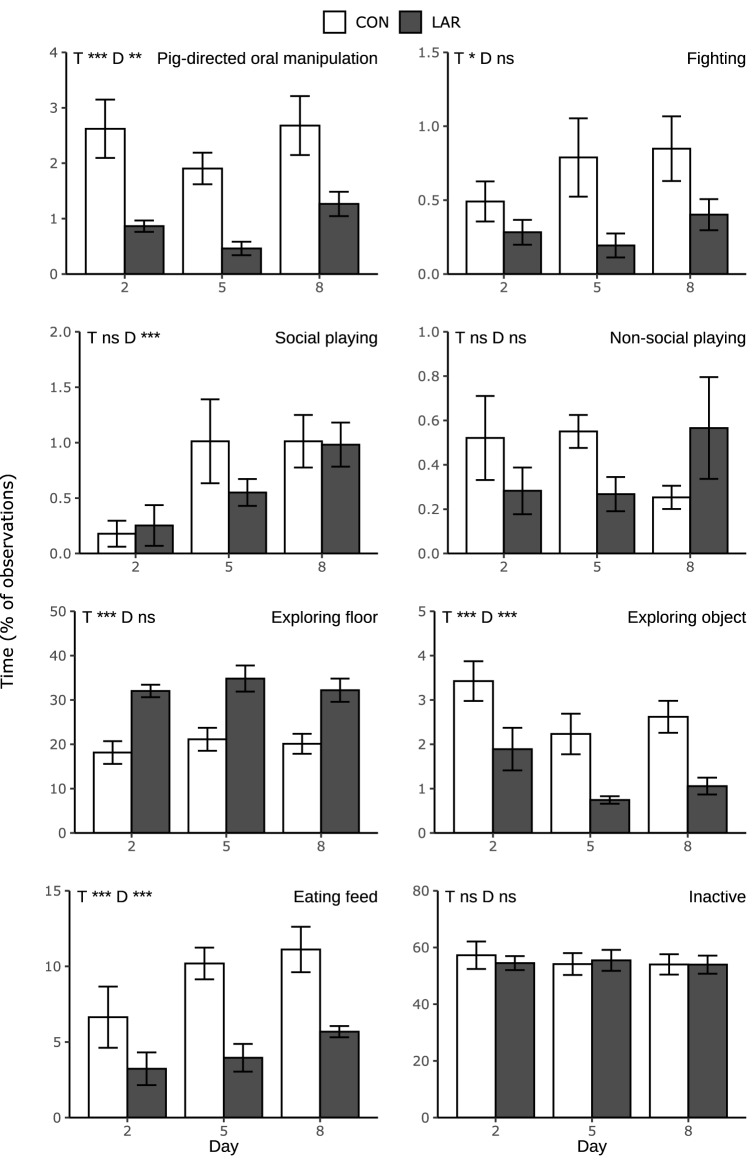
Behaviours observed in the home pen. Behavioural time budgets expressed as pen means ± SEM of pigs receiving wood shavings (CON) or black soldier fly larvae (LAR) twice a day. Effects of Treatment (T) and Day (D) are indicated as ***p < 0.001, **p < 0.01, *p < 0.05 and ns if p > 0.05. The Treatment x Day interaction was never significant and is therefore not shown.

Averaged over the three time points, LAR piglets spent less time on pig-directed oral manipulation (F(1,13) = 24.27, p < 0.001), fighting (F(1,13) = 6.92, p = 0.02), exploring objects (F(1,13) = 17.89, p < 0.001) and eating feed (F(1,13) = 23.89, p < 0.001), and they spent more time on exploring the floor (F(1,13) = 29.14, p < 0.001) than the CON piglets. The time spent inactive (F(1,13) = 0.02, p = 0.89), social playing (F(1,13) = 0.39, p = 0.54), and non-social playing (F(1,13) = 0.09, p = 0.77) did not differ between treatments. Considering the effect of day, the time spent on pig-direct oral manipulation varied over time (F(1,78) = 5.13, p = 0.01), with the lowest levels occurring on day 5 as compared to day 2 and 8 after weaning. The time spent on exploring objects decreased over time (F(1,78) = 9.33, p < 0.001) and the time spent on social play (F(1,78) = 18.18, p < 0.001) and eating feed (F(1,78) = 7.93, p < 0.001) increased over time. The time spent fighting (F(1,78) = 2.43, p = 0.09), non-social playing (F(1,78) = 0.00, p = 1.00), exploring the floor (F(1,78) = 1.90, p = 0.16) and being inactive (F(1,78) = 0.45, p = 0.64) were not influenced by day (Fig. 1).

### Novel environment and novel object test

The one retained factor of the novel environment test had high positive loadings for standing, freezing and vocalizations, and high negative loadings for exploring the environment and moving, therefore this factor will be designated as “fearful” (Table 3). Treatment did not influence the pigs’ scores for this factor (CON: 0.02 ± 0.20, LAR: 0.03 ± 0.28, F(1,13) = 0.02, p = 0.90).

The one retained factor of the novel object test had high positive loadings for freezing and the latency to explore the object, and high negative loadings on time spent moving and exploring the object, and this factor will be designated as “neophobic” (Table 3). Here, treatment influenced piglet scores, where CON piglets scored higher on the “neophobic” factor than LAR piglets (CON: 0.43 ± 0.27, LAR: − 0.39 ± 0.25, F(1,13) = 5.30, p = 0.04).

### Performance

Performance parameters measured over the experimental period are shown in Table 4. Treatment did not affect average daily gain (F(1,13) = 0.07, p = 0.79) and final weight (F(1,13) = 0.07, p = 0.79) of the piglets, and it also did not influence their feed efficiency (based on dry matter intake including BSFL, F(1,12) = 2.64, p = 0.13) and energy efficiency (based on dry matter intake including BSFL, F(1,12) = 0.71, p = 0.42)). Dry matter intake excluding BSFL over the whole experimental period was lower in LAR piglets than in CON piglets (F(1,12) = 6.35, p = 0.03). Detailed analysis of the separate periods for which feed intake was determined showed that dry matter intake excluding BSFL was lower in LAR piglets than in CON piglets during day 4–7 (F(1,13) = 5.57, p = 0.03) and during day 7–11 (F(1,12 = 10.18, p = 0.01, Supplementary Table S2). Treatment did not influence the dry matter intake including BSFL (F(1,12) = 0.06, p = 0.82) or the total energy intake including BSFL (F(1,12) = 2.31, p = 0.15).

**Table 4:** Average daily gain, body weight, dry matter intake (excluding and including black soldier fly larvae (BSFL)), energy intake (including BSFL), feed efficiency, and energy efficiency of pigs receiving wood shavings (CON) or black soldier fly larvae (LAR) twice a day.

Measure	Period	CON	LAR	p
Average daily gain (g/pig/day)	d0–d11	208 ± 17	214 ± 14	0.79
Body weight (g)	d0	7325 ± 47	7390 ± 64	0.79
	d11	9611 ± 173	9748 ± 189	0.79
Dry matter intake excl. BSFL (g/pig/day)	d0–d11	215 ± 14	168 ± 11	**0.03**
Dry matter intake incl. BSFL (g/pig/day)	d0–d11	215 ± 14	211 ± 11	0.82
Energy intake incl. BSFL (MJ NE/pig/day)	d0-d11	4.7 ± 0.3	5.3 ± 0.2	0.15
Feed efficiency (body weight gain (g/pig)/dry matter intake incl. BSFL (g/pig))	d0–d11	0.92 ± 0.04	1.01 ± 0.04	0.13
Energy efficiency (body weight gain (g/pig)/energy intake incl. BSFL (MJ NE/pig))	d0–d11	84.2 ± 3.5	79.8 ± 3.1	0.42

Data are expressed as means ± SEM.

Significant p-values are presented in bold.

### Faecal consistency and dry matter content

Faecal consistency scores of 3 and 4 were rare (< 2%), and as only these scores indicate diarrhoea, the faecal consistency scores were not further analysed. The faecal dry matter content on day 4 and 7 was not influenced by treatment (Day 4, CON: 31.6 ± 1.7%, LAR: 28.7 ± 1.4%, F(1,13) = 1.14, p = 0.30, Day 7, CON: 28.6 ± 3.4%, LAR: 27.7 ± 1.7%, F(1,13) = 0.02, p = 0.89).

## Discussion

In this study, we investigated the potential of using live black soldier fly larvae (BSFL) as edible enrichment for piglets post-weaning. Providing a small amount of live BSFL twice a day increased floor-directed exploration, while it decreased object-directed exploration, pig-directed oral manipulation, fighting, and time spent eating pellets. In addition, a factor analysis suggests that LAR piglets were less neophobic, i.e. fearful of a novel object, than CON piglets when this object is introduced to them in an unfamiliar environment. Daily consumption of a small amount of live BSFL reduced pellet intake, but total feed and net energy intake (including BSFL), average daily gain, feed efficiency, energy efficiency, and faecal consistency were not affected by live BSFL provisioning.

In line with our expectations, live BSFL provisioning redirected exploration and manipulation behaviours away from objects and conspecifics and towards the floor where the larvae were provided, while simultaneously decreasing aggression. These effects occurred over the whole day despite the larvae being only transiently present each day and indicate that temporary access to live BSFL can benefit piglet welfare. In the ethogram applied in the current study, pig-directed oral manipulation included nosing of pen mates, which does not necessarily negatively impact piglet welfare. However, under barren (i.e. without bedding and with limited space) commercial conditions, pig-directed oral manipulation often accumulates into damaging behaviours that are harmful to pigs, such as tail biting^4,17,43^. Additionally, post-weaning manipulation behaviours and aggression tend to be more common in barren commercial conditions compared to our experimental setting^17,44,45^. Therefore, the observed welfare benefits associated with live BSFL provisioning are likely amplified in commercial pig husbandry conditions, and this merits investigation in future studies.

As live BSFL possess many features interesting for pigs^32,33^, they likely provided ample exploration opportunities to satisfy the piglet’s exploratory needs. By promoting exploration, the motivation to perform other behaviours such as oral manipulation behaviours directed at pen mates may be decreased. Moreover, providing enrichment can have stress-reducing effects on pigs^14^, and as such, the presence of larvae may have attenuated the piglet’s weaning stress. This could have shifted their behavioural repertoire towards more favourable behaviours and reduced aggressive behaviours. In line with our results, increased exploration and reduced harmful social behaviour were also found in studies using peat, straw, or a complex environment with increased space and a multitude of enrichment materials^4,17,25^. However, while these studies also observed increased play behaviour in enriched pigs, live BSFL provisioning did not affect social and non-social play. Possibly, the elevated level of exploratory behaviour direct towards the BSFL reduced the motivation to perform other behaviours including (social) play. Also, in the current study piglets were housed in relatively small groups with 2 piglets per pen as compared to 4–11 piglets per pen in the previous studies, and facilitation of social play was therefore relatively low. On the other hand, all CON and LAR piglets in the current study had access to bedding, which can already promote play as compared with barren environments^46^. The influence of the experimental conditions may have outweighed any additional effects of live BSFL provisioning on play behaviour, and differential effects on play behaviour may be found under commercial conditions.

Besides studying home-pen behaviour, behavioural responses to novel situations were determined and analysed with a factor analysis. It can be speculated that piglets with reduced neophobic responses can better cope with novel situations such as changes to their environment. The factor resulting from the analysis of the novel object test data had high positive loadings on the time spent freezing and the latency to explore the unfamiliar object, and high negative loadings on time spent moving and exploring the object. Previous studies indicated that relatively slower and lesser exploration of a (novel) object and being less active in unfamiliar situations can be associated with increased fearfulness and/or anxiety^47–49^, and an alert standing posture, i.e. freezing, is often exhibited in response to aversive situations^50^. Donald et al. found a factor with similar behavioural components and loadings for piglets participating in an open field test in which novel objects were present, and concluded that this factor mainly reflected the degree of neophobia, as the fear-related behaviours occurred in response to novelty^51^. Likewise, the factor found in our study is assumed to reflect the level of neophobia, and as LAR piglets scored lower on this factor compared to CON piglets, LAR piglets are assumed to be less neophobic. The transient presence of live larvae is therefore sufficient to decrease neophobia. This may indicate that the feedback provided by tasting and eating larvae enhances the positive associations with novelty enough to compensate for the occasional absence of larvae. In line with our results, long-term access to live BSFL decreased broiler fearfulness, as shown by a decreased amount of time spent in tonic immobility^52^. Similarly, previous studies found that housing pigs with marginally increased space and more toys elevated exploration of a novel object^53^, and providing hanging ropes together with hanging rubber tyre tubes reduced the latency to approach a person^15^.

It should be noted that reduced neophobic responses by piglets that were given larvae were only observed in the novel object test, and not in the preceding novel environment test. The factors retained in this test had, similarly to the novel object test, a high negative loading for moving and a high positive loading on freezing. Additionally, this factor had a high negative loading for time spent on exploring the environment, and high positive loadings for standing and the frequency of vocalizations. In a previous study, pigs which had received fear-reducing drugs spent more time on exploring their environment and less time on vocalizing compared to saline-treated pigs^51^, indicating that these behaviours are also related to fearfulness. Taken together, the factor retained from the novel environment test is assumed to reflect general fearfulness and/or anxiety. The observation that scores for this factor were not affected by treatment likely relates to the stimulus-specific responses of piglets towards novelty. This was demonstrated by Hemsworth et al. who found that piglets that received objects in their home pen were more willing to explore a novel object compared to piglets that had received human contact in their home pen^11^. The absence of any observed behaviour difference in the novel environment test could therefore be due to the absence of novel items in this test. A novel object test was previously found to cause more fear-related behaviour and higher serotonergic responses compared to a novel environment test^49^, and the lower fear response prompted by a novel environment might not be sufficient to observe any treatment effects on fearfulness. While our results demonstrate the potential of live BSFL provisioning to reduce neophobia in piglets, further research is required to assess whether also general fearfulness is affected.

Some studies have found a link between increased exploration behaviour and increased feed intake pre-weaning^54^ and post-weaning^55^. As live BSFL provisioning increased exploratory behaviours, it was expected to also increase feed intake, and potentially increase the time spent eating pellets. In contrast, LAR piglets spent less time eating pellets than CON piglets, and pellet consumption during day 4–11 was reduced in LAR piglets. The main reason that pellet consumption was reduced by live BSFL provisioning appears to be that consumption of live BSFL sufficiently fulfilled the pigs’ motivation to eat. In a previous study in which a range of dietary options were available pre-weaning, consumption of the more palatable feed items was preferred^22^, and the high palatability of BSFL likely caused a similar preference and high consumption. Including BSFL meal in pig diets has previously had positive^56^ or neutral^57,58^ effects on pig growth, while the impact of consuming whole, live BSFL on piglet performance has, to our knowledge, not been studied. The absence of any effect of whole live BSFL consumption on total dry matter intake, net energy intake, feed efficiency, energy efficiency and average daily gain illustrates the nutritional equivalence of BSFL to feed on a dry matter basis in the current experimental setting. This finding highlights the potential nutritional value of BSFL for pigs, as suggested previously^28,31^. It is important to note that availability of BSFL in the current study was restricted to a fixed daily portion, and ad libitum provisioning could differentially influence piglet performance. Immediately post-weaning, when solid feed intake is generally low^2^, ad libitum BSFL provisioning might boost piglet performance by increasing nutrient intake. This, in turn, likely supports good intestinal integrity and growth^59^, warranting future investigation. Furthermore, when a substantial part of the diet is replaced with larvae, it will be necessary to adjust the nutrient composition of the regular feed to assure adequate pig performance. Optimizing a pig diet including larvae requires further research into the complete nutrient composition and digestibility of BSFL.

The piglets’ faecal dry matter content was not affected by live BSFL consumption. The occurrence of diarrhoea, indicated by a faecal consistency score of 3 or 4^41^, was very low in both treatment groups. This could be the results of the wood shavings present in all pens, which are known to reduce the number of days pigs have diarrhoea and benefit the overall faecal consistency score^60^. Under commercial conditions, diarrhoea is more common, for example due to the reduced feed intake and increased stress around weaning (as reviewed by^59^). Investigating live BSFL provisioning in this setting is recommended to determine their effect on post-weaning diarrhoea.

To conclude, post-weaning live BSFL provisioning had beneficial effects on piglet behaviour, by facilitating exploration behaviours and reducing the need to orally manipulate objects and pen mates. The presence of larvae also reduced neophobic responses towards a novel object. The performance of piglets that consumed a small amount of larvae was maintained, as their total feed and net energy intake, feed efficiency, energy efficiency, and average daily gain did not differ from that of control piglets. Under barren commercial conditions, larvae provisioning has the potential to further benefit piglet welfare, though this remains to be confirmed by future studies. These studies should also consider appropriate larvae provisioning methods under commercial conditions, as scattering larvae on slatted floors is impractical.

## Supplementary Information

Supplementary Information.
