# Supplementary material for: Providing live black soldier fly larvae (Hermetia illucens) improves welfare while maintaining performance of piglets post-weaning
Source: Sci Rep. 2021 Apr 1;11:7371. doi: 10.1038/s41598-021-86765-3 (PMC8016837; doi:10.1038/s41598-021-86765-3)
Supplement: Supplementary file 1 — Supplementary Information. [file 41598_2021_86765_MOESM1_ESM.docx]

Providing live black soldier fly larvae (*Hermetia illucens*) improves welfare while maintaining performance of piglets post-weaning

Allyson F. Ipema^1^*, Eddie A.M. Bokkers^2^, Walter J.J. Gerrits^3^, Bas Kemp^1^ & J. Elizabeth Bolhuis^1^

^1^ Adaptation Physiology Group, Department of Animal Sciences, Wageningen University & Research, P.O. Box 338, 6700 AH Wageningen, The Netherlands

^2^ Animal Production Systems Group, Department of Animal Sciences, Wageningen University & Research, P.O. Box 338, 6700 AH Wageningen, The Netherlands

^3^ Animal Nutrition Group, Department of Animal Sciences, Wageningen University & Research, P.O. Box 338, 6700 AH Wageningen, The Netherlands

*Correspondence and requests for materials should be addressed to A.I. (email: allyson.ipema@wur.nl).

*Keywords:* Pig, Black soldier fly larvae, Enrichment, Weaning, Behaviour, Fearfulness

**Supplementary tables**

**Table S1.** Analysed chemical composition of back soldier fly larvae (BSFL). Chemical composition analysis via standardized protocols (DM 10032, Protein 10005, Fat 10112, Calcium and Phosphorus 10040).

|  | **BSFL** |
| --- | --- |
| Dry matter (DM, g/kg) | 350.7 |
| ME (MJ/kg of DM) | 19.5 |
| Crude protein (g/kg of DM) | 419 |
| Crude fat (g/kg of DM) | 371 |
| Calcium (g/kg of DM) | 6.3 |
| Phosphorus (g/kg of DM) | 6.8 |

**Table S2**. Average daily gain and dry matter intake (excluding and including black soldier fly larvae (BSFL)) per period of pigs receiving wood shavings (CON) or black soldier fly larvae (LAR) twice a day. Data are expressed as means ± SEM. Significant p-values are presented in bold.

| **Measure** | **Period** | **CON** | **LAR** | **F-statistic and df** | **P** |
| --- | --- | --- | --- | --- | --- |
| Average daily gain (g/pig/day) | d0-d1 | -240 ± 22 | -188 ± 32 | F(1,13) = 1.65 | 0.22 |
|  | d1-d4 | 144 ± 38 | 133 ± 23 | F(1,13) = 0.06 | 0.81 |
|  | d4-d7 | 242 ± 34 | 204 ± 31 | F(1,13) = 0.62 | 0.45 |
|  | d7-d11 | 342 ± 15 | 384 ± 36 | F(1,13) = 1.08 | 0.32 |
| Dry matter intake excl. BSFL (g/pig/day) | d0-d1 | 13 ± 3 | 8 ± 3 | F(1,13) = 1.59 | 0.23 |
|  | d1-d2 | 58 ± 26 | 43 ± 21 | F(1,13) = 0.20 | 0.66 |
|  | d2-d4 | 120 ± 23 | 101 ± 14 | F(1,13) = 0.52 | 0.48 |
|  | d4-d7 | 220 ± 27 | 136 ± 23 | F(1,13) = 5.57 | **0.03** |
|  | d7-d11 | 367 ± 14 | 297 ± 14 | F(1,12) = 10.18 | **0.01** |
| Dry matter intake incl. BSFL (g/pig/day) | d0-d1 | 13 ± 3 | 21 ± 3 | F(1,13) = 2.87 | 0.11 |
|  | d1-d2 | 58 ± 26 | 69 ± 21 | F(1,13) = 0.11 | 0.75 |
|  | d2-d4 | 120 ± 23 | 134 ± 14 | F(1,13) = 0.24 | 0.63 |
|  | d4-d7 | 220 ± 27 | 188 ± 23 | F(1,13) = 0.78 | 0.39 |
|  | d7-d11 | 367 ± 14 | 350 ± 14 | F(1,12) = 0.62 | 0.45 |
